# Comparative outcomes of laparoscopic and robotic colorectal cancer surgery in the NHS: real-world evidence from sequential adoption of Versius and da Vinci Xi

**DOI:** 10.1007/s11701-026-03186-y

**Published:** 2026-02-11

**Authors:** Samuel Massias, Qamil Pajaziti, Arian Arjomandi Rad, Bibechan Thapa, Bhamini Vadhwana, Lillian Reza, Vanash Patel

**Affiliations:** 1 Department of Surgery, West Hertfordshire Teaching Hospitals NHS Trust, Watford General Hospital, Vicarage Road, Watford, WD18 0HB UK; 2 Department of Surgery and Cancer, Imperial College London, St Mary’s Hospital, 10th Floor QEQM Building, London, W2 1NY UK; 3 Department of Cardiothoracic Surgery, Oxford University Hospitals NHS Foundation Trust, Headley Way, Headington, Oxford, OX3 9DU UK

**Keywords:** Colorectal surgery, Laparoscopic surgery, Robotic-assisted surgery, Training, Learning curves

## Abstract

Multiple robotic systems are now available for colorectal cancer surgery, yet comparative real-world evidence to guide NHS adoption remains limited. This study compared perioperative, oncological, and learning-curve outcomes for laparoscopic, Versius (CMR), and da Vinci Xi (dV) resections. A single-centre evaluation included elective colorectal cancer resections between November 2021 and May 2025 using laparoscopy, CMR or dV. Primary outcomes were length of stay (LOS) and operative time; secondary outcomes included lymph-node yield and Clavien–Dindo ≥ 2 complications. Analyses used non-parametric tests, Bonferroni-adjusted comparisons and multivariable regression. Learning curves were assessed with rolling means, LOWESS and CUSUM. A total of 290 patients were included: laparoscopy (n = 85), CMR (n = 103), and dV (n = 102). Median LOS was 5, 5 and 4 days respectively (p < 0.001) with dV significantly shorter than laparoscopy after adjustment (− 2.33 days; 95% CI − 3.78 to − 0.88). Risk-adjusted analysis of prolonged LOS (> 5 days) showed lower odds with dV versus CMR (OR 0.45, 95% CI 0.23–0.85; p = 0.002), with adjusted probabilities of 22.1% for dV, 37.5% for CMR and 49.0% for laparoscopy. CMR had longer adjusted operative times than laparoscopy (+ 37.2 min; p = 0.001). Lymph-node yield was highest with dV (median 25.5 vs. 22; p = 0.007), confirmed in adjusted analysis (+ 4.0 nodes; p = 0.004). Major complications were similar; CMR rectal cases had higher unadjusted rates. Learning curves showed operative time reduction for both robotic systems, with earlier plateauing for dV. Both robotic platforms were safe and oncologically equivalent; however, dV demonstrated shorter LOS, higher lymph node yield, and a more favourable learning curve.

## Introduction

Robotic-assisted colorectal surgery has rapidly reshaped the landscape of minimally invasive care, offering enhanced dexterity, improved ergonomics, and highly precise instrumentation beyond what conventional laparoscopy can provide [1–3]. Yet despite these advantages, the substantial capital and maintenance costs—and the ongoing uncertainty around comparative clinical value—remain barriers to widespread NHS adoption [4, 5]. As more trusts implement robotic platforms, robust, real-world comparative evidence is essential. High-quality analyses of perioperative outcomes, complications, oncological quality, and recovery trajectories are critical to inform investment decisions, optimise implementation, and guide national policy for sustainable robotic expansion [6–9]. 

In the UK, the adoption of new surgical technologies increasingly requires alignment with the National Institute for Health and Care Excellence (NICE) Early Value Assessment (EVA) framework, which provides rapid, provisional guidance for promising but high-cost innovations. EVA emphasises the generation of real-world evidence on safety, clinical effectiveness, workflow impact, and cost-effectiveness before widespread NHS rollout. Robotic surgery, with its substantial capital and maintenance investment, sits squarely within this remit. Evaluating outcomes across emerging systems—alongside conventional laparoscopy—is therefore essential to support responsible implementation, optimise training and service delivery, and determine whether new technologies demonstrate sufficient value to justify broader NHS adoption [10–12]. 

Against this backdrop, the global soft-tissue robotics market is expanding rapidly. While the da Vinci platform remains the most established and widely adopted system worldwide, the past decade has seen the introduction of multiple new-generation robotic platforms into clinical practice [10, 13, 14]. These include CMR Surgical’s system, Medtronic’s Hugo RAS, MicroPort’s Toumai platform, Medicaroid’s Hinotori system, and SS Innovations’ SSi Mantra system, all of which are now in clinical use across various countries [15]. Additional platforms continue to advance toward broader adoption as technological diversification accelerates. This proliferation of robotic systems highlights the pressing need for rigorous outcome data to guide procurement, optimise training pathways, and support coherent long-term adoption strategies. Without clear evidence of clinical and economic value, health systems risk fragmented implementation and inequitable access across regions [10, 15]. 

Within this evolving landscape, West Hertfordshire Teaching Hospitals (WHTH) has uniquely implemented two distinct robotic platforms—Cambridge Medical Robotics Versius (CMR) and the Intuitive da Vinci Xi (dV) - within a high-volume NHS colorectal cancer service. This staged adoption, supported by consistent perioperative pathways, structured training, and multidisciplinary governance, provides a robust setting in which platform-level performance can be directly compared within a single institution. Evaluating outcomes across these systems, alongside our historical laparoscopic practice, offers an opportunity to generate high-quality real-world evidence aligned with national EVA priorities. This analysis aims to clarify the relative clinical performance, safety profile, and perioperative value of each platform, providing data to guide future procurement, training, and service configuration both locally and across the wider NHS [10].

## Methods

### Ethics approval

This project was not considered by the Health Research Authority (HRA). Therefore, HRA research ethics approval was not required for this study. The service evaluation was reviewed and approved by the information governance team and clinical lead for surgery at the WHTH before its commencement.

### Context

WHTH is a high-volume surgical centre that commenced a phased implementation of the CMR in 2022 across multiple specialties, including colorectal, upper gastrointestinal, urology, and gynaecology. Six colorectal consultants were trained on the CMR platform and began performing bowel cancer resections between July 2022 and April 2024, completing more than 100 procedures during this period [7]. In 2024, WHTH acquired the dV system, and four colorectal consultants were subsequently trained in its use, three of whom had prior experience operating with the CMR system. Since April 2024, colorectal resections at the Trust have been performed using the dV platform [8]. Before 2022, all surgeons involved in this study were fully trained and experienced in conventional laparoscopic bowel cancer resections.

Patients who underwent elective bowel cancer resection using laparoscopy, CMR or dV were included in this analysis. Recruitment periods were as follows: November 2021 to July 2022 for the laparoscopic cohort, July 2022 to August 2023 for the CMR cohort, and April 2024 to May 2025 for the dV cohort. Clinical data were extracted from electronic patient records and included patient demographics, diagnosis and staging, preoperative comorbidities, operative details (procedure type and duration), length of hospital stay, estimated blood loss, analgesic requirements, histological findings, and perioperative and postoperative complications.

Data prior to November 2021 could not be collected because Cerner Millennium was implemented at that time, and all data for this study were obtained from Cerner to ensure accuracy and consistency. Data extraction was performed by PQ and SM, and all data were independently checked for accuracy by VP. As our institution is a teaching hospital, all consultants operating on CMR or dV completed the company training pathway before undertaking independent cases, including an initial phase of proctored cases (included in this series). Once beyond their learning curve, consultants began training senior trainees through our structured robotic programme, with trainee involvement always under direct supervision.

### Statistical analysis

Continuous variables were summarised as median with interquartile range (IQR) and, where helpful, mean with standard deviation (SD). Categorical variables were reported as counts and percentages. Between-group comparisons of continuous outcomes (length of stay, total operating time, lymph-node yield) across the three surgical approaches (laparoscopy, CMR and dV) were performed using the Kruskal–Wallis test. Where an overall difference was detected, pairwise comparisons were undertaken using Mann–Whitney U tests with Bonferroni correction for multiple testing. Categorical outcomes (conversion to open surgery, anastomotic leak, unplanned ICU admission, transfusion, and Clavien–Dindo complication categories) were compared using χ² tests or Fisher’s exact test where expected cell counts were small. Baseline balance between groups was assessed using standardised mean differences (SMDs), with values ≥ 0.1 taken to indicate potential imbalance.

To explore learning effects, we constructed learning-curve plots of total operating time against chronological case number. For each platform, 5-case rolling means of operating time were plotted alongside locally weighted scatterplot smoothing (LOWESS) curves to illustrate trends over time in the whole cohort and in colon and rectal cancer subgroups. In addition, cumulative sum (CUSUM) plots of operative time were generated for all surgeons combined and for individual high-volume surgeons to visualise changes in performance relative to each surgeon’s mean operative time over successive cases.

Multivariable analyses were used to estimate associations between surgical approach and key outcomes after adjustment for case-mix. Binary outcomes (conversion to open surgery, anastomotic leak, unplanned ICU admission, transfusion, and Clavien–Dindo grade ≥ 2 within 30 days) were modelled using logistic regression, with laparoscopy as the reference category. Continuous outcomes (length of stay, total operating time and lymph-node yield) were modelled using linear regression. All models were adjusted for prespecified covariates including age, sex, body mass index (BMI), ASA grade, Charlson Comorbidity Index (CCI), CR-POSSUM predicted mortality, pathological stage group and type of resection. In a prespecified exploratory analysis, prolonged length of stay was modelled as a binary outcome (LOS > 5 days vs. ≤ 5 days) using logistic regression with the same covariates plus tumour site (colon vs. rectal) and malignancy status. Regression results are presented as odds ratios (ORs) or mean differences with 95% confidence intervals (CIs). All tests were two-sided and a p-value < 0.050 was considered statistically significant. Analyses were performed using SPSS (version 29, IBM Corp.) and R (version 4.4.0). Complete-case analysis was used throughout; no imputation was performed for missing data.

## Results

### Baseline characteristics

We included 290 colorectal cancer resections, comprising 85 laparoscopic cases (55 colon, 64.7%; 30 rectal, 35.3%), 103 CMR cases (64 colon, 62.1%; 39 rectal, 37.9%), and 102 dV cases (66 colon, 64.7%; 36 rectal, 35.3%). Overall, 56.6% of patients were male (laparoscopy 55.3%, CMR 59.2%, dV 54.9%). Median age was similar across groups, with values of 70 [62–77] years for laparoscopy, 70 [58–78] for CMR, and 69 [60.3–74.8] for dV, and only a small imbalance (SMD 0.11). BMI was modestly higher in the CMR cohort (27.35 [24.83–31.65] kg/m²) compared with laparoscopy (27.25 [24.6–29.0]) and dV (26.8 [24.2–30.3]), reflected by an SMD of 0.30.

CCI was comparable, with medians of 5 [4–5] in the laparoscopy group and 5 [4–6] in both CMR and dV (SMD 0.29). Physiologic risk (CR POSSUM) showed the greatest imbalance (max SMD 0.49), with median [IQR] values of 17 [16–19] in laparoscopy, 17 [14.5–18] in CMR, and 16 [15–17] in dV. ASA class was predominantly II–III across all groups, and both operative procedure type and postoperative pathological stage were broadly similar, with stage I–III disease accounting for the majority of cases.

Given the observed imbalances, particularly in CR POSSUM, BMI, and CCI, all comparative models were prespecified to adjust for age, sex, BMI, ASA, CCI, CR POSSUM, pathological stage group, and operation type.

### Primary outcomes

#### Length of stay (LOS)

Median [IQR] LOS was 5 [4–7] days for laparoscopy, 5 [4–7] for CMR, and 4 [2–5] for dV (*p* < 0.001). Pairwise testing showed dV was shorter than laparoscopy (adjusted *p* < 0.001) and shorter than CMR (adjusted *p* < 0.001). In multivariable models adjusted for age, sex, BMI, ASA, CCI, CR POSSUM, stage and operation type, dV was associated with a shorter LOS compared with laparoscopy, with an adjusted difference of minus 2.33 days (95% CI minus 3.78 to minus 0.88, *p* = 0.002). CMR showed no difference compared with laparoscopy (minus 0.18 days, 95% CI minus 1.62 to 1.27, *p* = 0.808).

#### Operating time

**Fig. 1:**
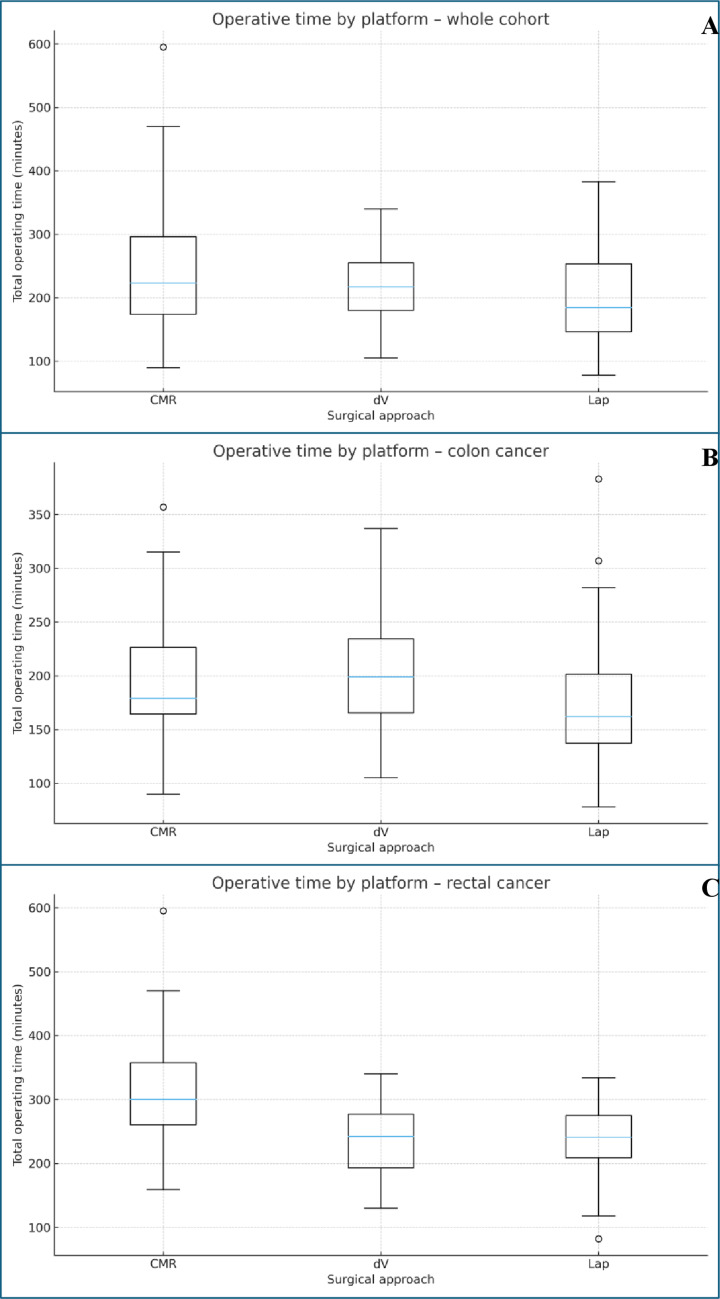
Operative time by platform (**A**) whole cohort, (**B**) colon cancer and (**C**) rectal cancer

Median operating time was 184.5 [146.2–253.5] min for laparoscopy, 223.0 [174.0–296.5] for CMR, and 217.0 [180.0–254.8] for dV (*p* = 0.005). Pairwise comparisons identified CMR as longer than laparoscopy (adjusted *p* = 0.004). Adjusted analyses confirmed CMR had a longer operating time than laparoscopy by plus 37.2 min (95% CI 15.8–58.7, *p* = 0.001). For dV versus laparoscopy, the adjusted difference was plus 17.2 min (95% CI minus 4.2 to 38.5, *p* = 0.114). Figure 1 shows operative time by platform.

### Secondary outcomes

#### Oncological outcomes

Median yields were 22 [15–28] for laparoscopy, 23 [17–29] for CMR, and 25.5 [20.2–32.0] for dV, with an overall difference (*p* = 0.007). Pairwise testing indicated dV was greater than laparoscopy (adjusted *p* = 0.010). Adjusted analysis showed dV retrieved plus 4.00 nodes compared with laparoscopy (95% CI 1.29–6.71, *p* = 0.004), while CMR was not different (plus 1.44 nodes, 95% CI minus 1.26 to 4.15, *p* = 0.295). R0 resection was achieved in 84/85 (98.8%) laparoscopic, 102/103 (99.0%) CMR, and 100/102 (98.0%) dV cases, with no statistically significant differences between platforms (*p* = 1.000 for laparoscopy vs. CMR; *p* = 1.000 for laparoscopy vs. dV; *p* = 0.620 for CMR vs. dV).

#### Conversion to open

Rates were 7.1% (6/85) for laparoscopy, 9.7% (10/103) for CMR, and 2.9% (3/102) for dV (overall *p* = 0.143). Adjusted odds versus laparoscopy were 1.46 (95% CI 0.37–5.77, *p* = 0.587) for CMR and 0.85 (0.17–4.23, *p* = 0.843) for dV.

#### Anastomotic leak

In this analysis, only resections with a primary anastomosis were included, including cases with a protective ileostomy. Anastomotic leaks were managed conservatively, radiologically, or surgically. Observed rates were 5.2% (3/58) for laparoscopy, 6.3% (5/79) for CMR, and 1.4% (1/74) for dV (overall *p* = 0.289). Adjusted odds ratios versus laparoscopy were 1.48 (95% CI 0.30–7.24, *p* = 0.625) for CMR and 0.22 (0.02–2.53, *p* = 0.223) for dV.

#### Unplanned ICU admission

Rates were 3.5% (3/85) for laparoscopy, 3.9% (4/103) for CMR, and 2.0% (2/102) for dV (overall *p* = 0.704). Adjusted odds ratios were 1.08 (95% CI 0.19–6.16, *p* = 0.931) for CMR and 0.59 (0.08–4.30, *p* = 0.604) for dV versus laparoscopy.

#### Transfusion

Rates were 10.6% (9/85) for laparoscopy, 8.7% (9/103) for CMR, and 4.9% (5/102) for dV (overall *p* = 0.334). Adjusted odds ratios were 0.92 (95% CI 0.27–3.08, *p* = 0.888) for CMR and 0.88 (0.23–3.32, *p* = 0.844) compared with laparoscopy.

#### Clavien Dindo grade 2 or higher complications within 30 days

Rates were 28.2% (24/85) for laparoscopy, 39.8% (41/103) for CMR, and 20.6% (21/102) for dV (overall *p* = 0.010). In adjusted models, neither robotic platform differed significantly from laparoscopy: CMR odds ratio 1.51 (95% CI 0.76–2.98, *p* = 0.240) and dV odds ratio 0.61 (95% CI 0.29–1.28, *p* = 0.193).

### Subgroup analysis

We performed a prespecified subgroup analysis stratified by tumour site (colon and rectal) using the same endpoints as in the overall cohort.

#### Colon cancer

For colon cancer (*n* = 185), the pattern of results was similar to the whole cohort: dV was associated with a shorter length of stay (median 3 [2–5] days) compared with both laparoscopy and CMR (each 5 [4–7] days; *p* < 0.001), whereas operating times were shortest with laparoscopy (162 [137.5–201.5] min), intermediate with CMR (179 [164.5–226.5] min), and longest with dV (199 [165.5–234.5] min; *p* ≈ 0.006). Lymph-node yield, R0 resection rates, conversion to open surgery, anastomotic leak, unplanned ICU admission, transfusion requirement, and Clavien–Dindo grade ≥ 2 complications did not differ materially between approaches in colon-only cases (all *p* > 0.050), although point estimates for conversion and complications favoured the da Vinci platform (Table 1).

#### Rectal cancer

In rectal cancer (*n* = 105), dV again showed a shorter length of stay (5 [4–6] days) compared with CMR and laparoscopy (each 6 [5–7] days; *p* ≈ 0.015). Operating time in rectal resections was substantially longer with CMR (300 [260.5–357.5] min) than with laparoscopy (241 [209–275] min) or dV (242 [193–277] min; *p* < 0.001). Lymph-node yield was highest for dV (median 25.5 [18–29.8]) vs. CMR (20 [15–26]) and laparoscopy (17.5 [12–24]; *p* ≈ 0.010). R0 resection rates, conversion to open surgery, anastomotic leak, unplanned ICU admission and transfusion were rare and comparable between groups (all *p* > 0.050); however, Clavien–Dindo grade ≥ 2 complications occurred more frequently with CMR (51.3%) than with laparoscopy (33.3%) and dV (22.2%; *p* ≈ 0.030), although these estimates are based on modest sample sizes and are unadjusted (Table 1).

**Table 1:** Comparative outcomes in colon and rectal cancers

Colon cancers	Laparoscopic *n* = 55	Versius *n* = 64	da Vinci Xi *n* = 66	*p* value
Median [IQR]				
Length of stay	5 [4–7]	5 [4–7]	3 [2–5]	*p* < 0.001
Operating time	162 [137.5–201.5]	179 [164.5–226.5]	199 [165.5–234.5]	0.006
Lymph node yield	22 [18–30]	24 [18–29.2]	25.5 [22–32.8]	0.200
% (n)				
R0 resection	100 (55)	100 (64)	99 (65)	0.404
Conversion to open	10.9 (6)	10.9 (7)	3 (2)	0.169
Anastomotic leak	3.8 (2/52)	6.8 (4/59)	1.8 (1/57)	0.396
Unplanned ICU admission	5.5 (3)	4.7 (3)	1.5 (1)	0.473
Transfusion	12.7 (7)	12.5 (8)	6.1 (4)	0.373
Clavien Dindo ≧ 2	25.5 (14)	32.8 (21)	19.7 (13)	0.232

Rectal cancers	Laparoscopic *n* = 30	Versius *n* = 39	da Vinci Xi *n* = 36	*p* value
Median [IQR]				
Length of stay	6.0 [5.0–7.0]	6.0 [5.0–7.5]	5.0 [4.0–6.0]	0.015
Operating time	241 [209–275]	300 [260.5–357.5]	242 [193–277]	*p* < 0.001
Lymph node yield	17.5 [12–24]	20 [15–26]	25.5 [18–29.8]	0.010
% (n)				
R0 resection	97 (29)	97 (38)	97 (35)	0.981
Conversion to open	0 (0)	7.7 (3)	2.8 (1)	0.235
Anastomotic leak	16.7 (1/6)	5 (1/20)	0 (0/17)	0.248
Unplanned ICU admission	0 (0)	2.6 (1)	2.8 (1)	0.664
Transfusion	6.7 (2)	2.6 (1)	2.8 (1)	0.626
Clavien Dindo ≧ 2	33.3 (10)	51.3 (20)	22.2 (8)	0.030

#### Operative time distributions by surgical platform

Box-and-whisker plots of operative time by platform (Fig. 1) demonstrated clear differences in efficiency and variability across the cohort. Overall, laparoscopic cases had the shortest median operative times, while CMR displayed longer median times with greater variability, particularly in rectal cancer cases. The dV platform had intermediate median operative times but the narrowest interquartile ranges, indicating more consistent operative performance. As expected, rectal resections showed longer and more dispersed operative times than colon resections across all platforms. Together, these distributions highlight that dV offers the most predictable operative performance, whereas CMR shows wider variation and higher operative times, especially for more complex pelvic procedures.

### Risk-adjusted LOS greater than 5 days

We performed a risk-adjusted analysis of prolonged length of stay (LOS > 5 days) using multivariable logistic regression including age, sex, BMI, ASA, CR-POSSUM predicted mortality, tumour site (rectal vs. colon) and malignancy status. Crude proportions of LOS > 5 days were 40.8% for CMR, 43.5% for laparoscopy and 23.0% for dV. After adjustment, dV was associated with significantly lower odds of LOS > 5 days compared with CMR (OR 0.45, 95% CI 0.23–0.85), whereas laparoscopy did not differ significantly from CMR (OR 1.67, 95% CI 0.81–3.44). The corresponding risk-adjusted probabilities of LOS > 5 days were 22.1% for dV, 37.5% for CMR and 49.0% for laparoscopy. An overall likelihood-ratio test for surgical approach was statistically significant (*p* ≈ 0.002), indicating that platform choice remained independently associated with prolonged LOS after adjustment for case mix.

### Learning-curve

#### Whole cohort

**Fig. 2:**
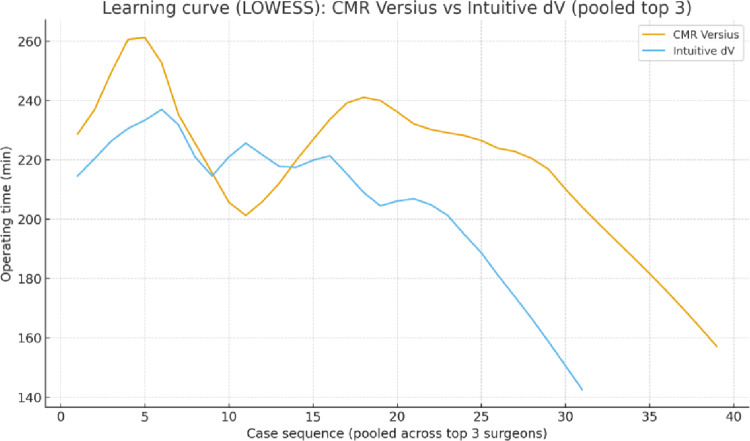
Learning curve CMR vs. dV (pooled top 3 surgeons)

Among the three highest-volume surgeons, both LOWESS-smoothed and rolling-mean plots showed early reductions in operating time on each robotic platform (Fig. 2). When pooled across surgeons, mean operating time decreased from 254.6 min in the first 10 CMR cases to 213.2 min after case 20 (Δ − 41.4 min), and from 218.7 min to 187.8 min for dV (Δ − 30.9 min).

At the individual surgeon level:

- Surgeon 1: dV operating time decreased from 221.0 to 182.6 min (Δ − 38.3), while CMR showed only a small change (238.9 → 235.6 min, Δ − 3.3).
- Surgeon 2: CMR operating time fell from 253.7 to 219.6 min (Δ − 34.1); dV increased slightly from 212.0 to 221.2 min (Δ + 9.2), reflecting limited early exposure.
- Surgeon 3: CMR decreased from 271.3 to 239.6 min (Δ − 31.7); dV increased from 223.1 to 238.8 min (Δ + 15.7), although this is based on a small series.

Regarding prior experience, Surgeons 1–3 had completed 41, 27, and 17 CMR cases, respectively, before performing their first dV case. Their mean operating times for the first 15 dV procedures were 217, 213, and 232 min, suggesting that previous robotic experience may have contributed to achieving acceptable early dV performance, although no formal causal analysis was undertaken.

#### Subgroup analysis

Learning-curve analyses restricted to the three highest-volume surgeons demonstrated clear reductions in operating time with increasing experience across both tumour-site subgroups. For colon resections, CMR operating time decreased from ~ 199 min in the early cases (≤ 10) to ~ 165 min in the later cases (> 20), while dV decreased from ~ 204 to ~ 164 min over the same intervals. For rectal resections, CMR operating time fell from ~ 384 min in the early cases (≤ 10) to ~ 276 min in the later cases (> 20), and dV decreased from ~ 249 to ~ 223 min.

#### Learning curve analysis

**Fig. 3:**
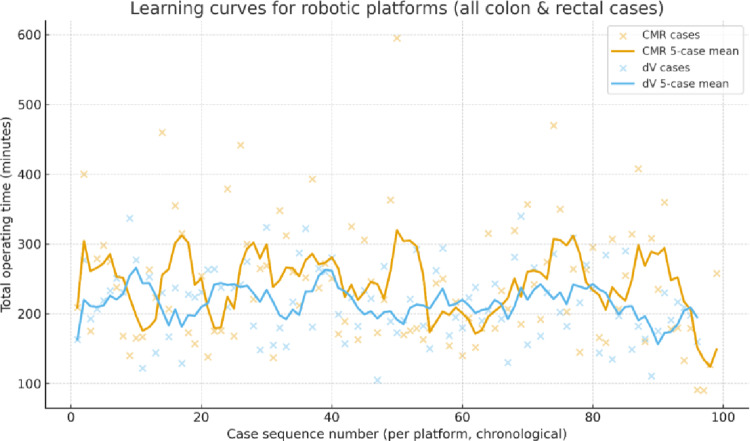
Learning curves for robotic platforms (whole cohort)

Learning-curve plots of total operative time against chronological case number demonstrated clear evidence of performance improvement for both robotic platforms (Fig. 3). Across the overall robotic cohort, 5-case rolling-mean curves for CMR and dV showed progressive reductions in operative time with increasing experience, consistent with an operative learning effect. The dV platform demonstrated a smoother, more consistent downward trajectory, with earlier plateauing at a lower operating-time range. CMR curves also declined over time but displayed greater variability, particularly during the early and mid-adoption phases. Taken together, these patterns indicate that proficiency was achieved with both systems, although the pace and stability of improvement appeared to differ between platforms.

**Fig. 4:**
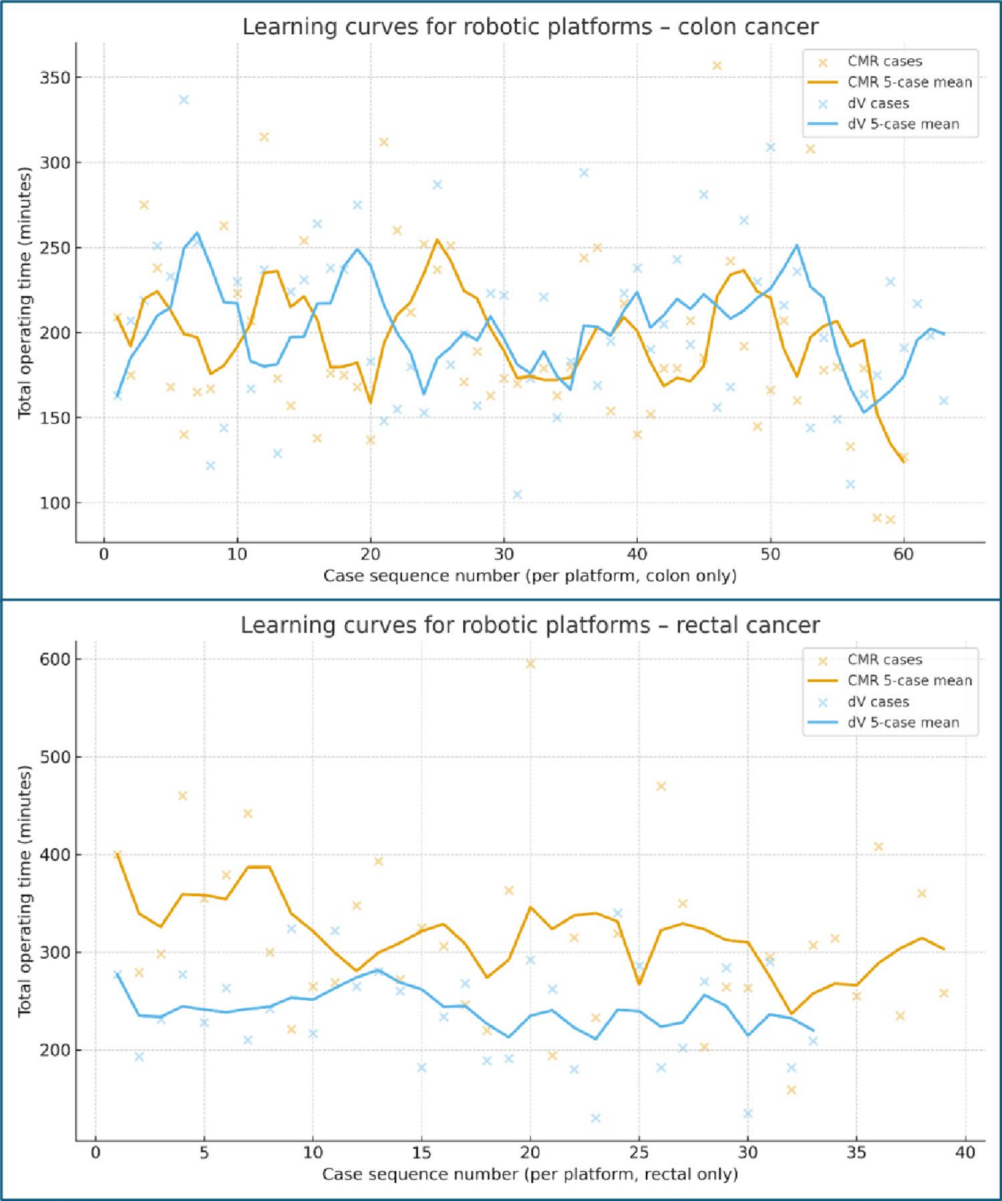
Learning curves for robotic platforms – colon and rectal cancers

When stratified by tumour site, similar trends were observed (Fig. 4). For colon cancer, operating times for both CMR and dV decreased with cumulative experience, but dV curves tended to stabilise earlier and at lower operative times, whereas CMR curves showed a more gradual descent and greater variability. In rectal cancer, the learning effect for CMR appeared particularly marked: early rectal CMR cases were associated with long operating times, followed by a substantial reduction as case numbers increased, indicative of a steeper learning curve. Rectal dV procedures started at comparatively lower operating times and demonstrated a more modest decline towards a stable plateau. These subgroup plots complement the subgroup summary statistics, reinforcing that operative efficiency improves over time for both platforms, with especially large gains seen for CMR in rectal resections.

#### CUSUM analysis of operative times

**Fig. 5:**
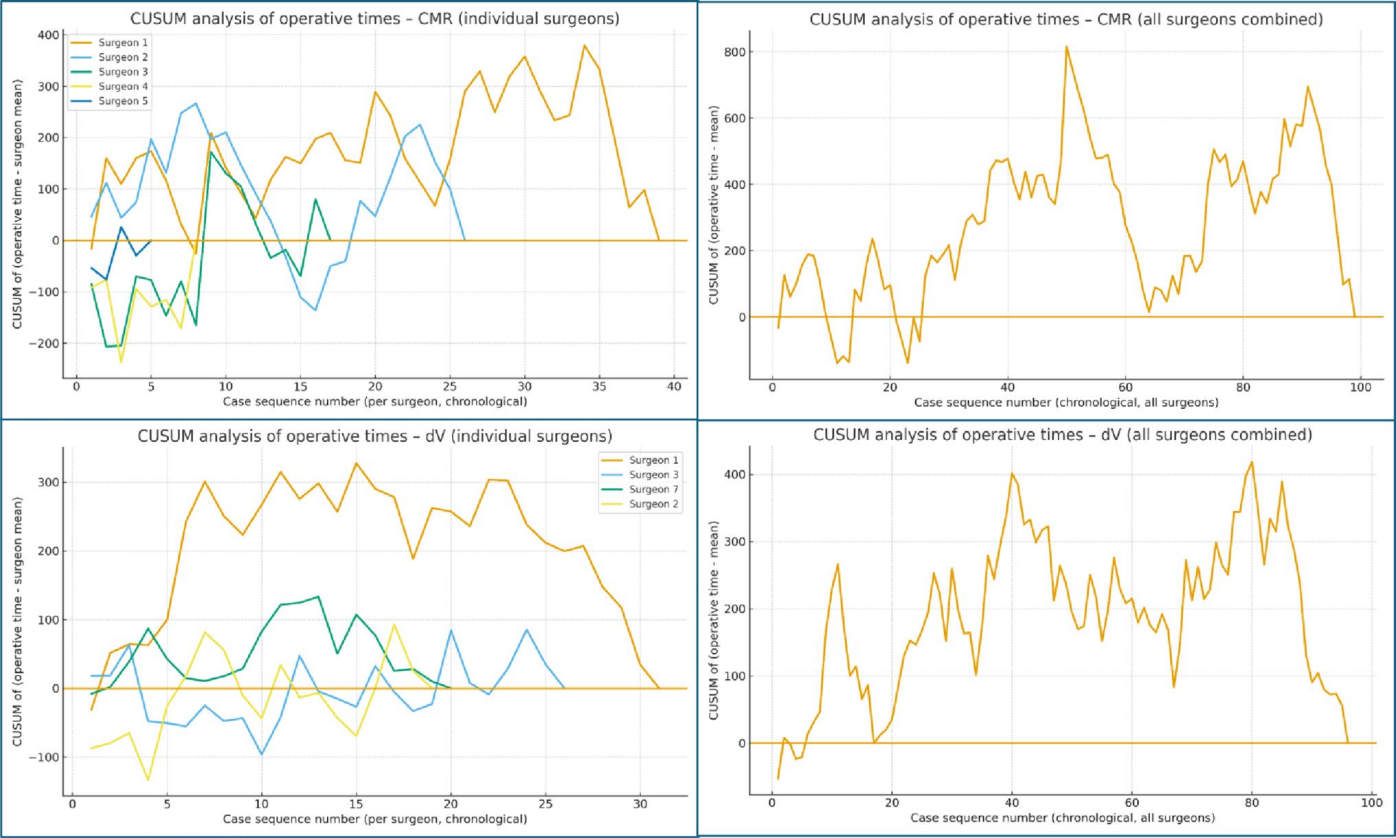
CUSUM analysis of operative times for CMR and dV platforms

CUSUM plots of operative time demonstrated learning effects for both robotic platforms at cohort and individual-surgeon level (Fig. 5). When all surgeons were analysed together, the CMR CUSUM curve initially showed a steady upward trajectory, indicating a run of cases with operative times above the overall mean, followed by a gradual flattening and subsequent downward trend as experience accumulated and operative times fell below the mean. The corresponding dV curve showed a shorter and less pronounced initial rise, with an earlier inflection point and more rapid progression to a stable or declining phase. This pattern is consistent with both platforms exhibiting operative learning, with dV achieving a “steady-state” performance after fewer cases and CMR showing a more extended early phase of above-average operative times.

Individual-surgeon CUSUM plots revealed marked heterogeneity in learning patterns (Fig. 5). For several surgeons, dV curves displayed only modest early increases followed by an early plateau or gradual decline, suggesting relatively rapid convergence towards each surgeon’s typical operative time. In contrast, CMR curves for the same surgeons more often showed larger early rises and more variable inflection points, with some displaying prolonged upward trends before flattening or falling. For other surgeons, CUSUM trajectories for both platforms were broadly similar, underlining that learning behaviour is partly surgeon-specific. Overall, the individual CUSUM curves reinforce the cohort-level findings by illustrating that, while learning occurs with both systems, the magnitude and timing of performance improvement differ between surgeons and platforms.

Taken together, these CUSUM analyses support the presence of operative learning curves for both CMR and dV, with a general pattern of higher operative times in early cases, followed by stabilisation and, in many instances, improvement over time. The steeper and more prolonged early increases observed in some CMR curves suggest a longer or more variable familiarisation phase for this platform in certain surgeons. However, these observations are descriptive and may be influenced by case selection, differences in prior robotic experience, and relatively small numbers of cases per surgeon, and therefore should not be interpreted as definitive evidence of intrinsic differences in adoptability between systems.

## Discussion

In this single-centre real-world evaluation of 290 colorectal cancer resections performed during sequential adoption of CMR and dV within a high-volume NHS service, we found that the dV platform was associated with significantly shorter length of stay, higher lymph node yield, and lower conversion and complication rates compared with both laparoscopy and CMR, although several differences did not reach statistical significance after risk adjustment. CMR demonstrated safe performance across all oncological and perioperative end points, but was associated with longer operating times compared with laparoscopy and dV, particularly in rectal cancer. These findings provide contemporary evidence to support platform selection, service configuration, and training strategy as NHS centres expand robotic colorectal surgery in line with the NICE EVA framework [16]. 

The most consistent finding across the whole cohort and tumour-specific analyses was the shorter length of stay observed with dV. This improvement persisted after adjustment for age, sex, BMI, ASA, CCI, CR POSSUM and pathological stage, suggesting that the benefit reflects perioperative performance rather than case-mix artefact. Virtual Hospital pathways were introduced during the robotic phase and may have contributed to shorter length of stay in dV cases. However, only a minority of patients were enrolled, and these were limited to colon cancer resections, so the pathway effect is likely modest. Importantly, even after adjustment, the reduction in prolonged stay (> 5 days) remained significant, supporting a genuine platform-related benefit that extends beyond pathway changes. Reduced length of stay with robotic platforms has been variably reported in previous meta-analyses [17], with many studies limited by selection bias, early-phase robotic adoption, and heterogeneity in perioperative pathways. Our data, collected within a mature enhanced-recovery programme and with consistent postoperative protocols across all three platforms, may support dV as an enabler of more rapid postoperative recovery [15]. 

In terms of operative performance, CMR demonstrated significantly longer operating times compared with laparoscopy and dV, particularly for rectal cancer where early cases often exceeded 300 min. A marked learning effect was evident: operating time for CMR fell by more than 100 min between early and later rectal cases among high-volume surgeons. This is consistent with published early-adoption studies of new robotic platforms, many of which describe prolonged operative times during the initial phase of robotic implementation [18]. In contrast, operating times for dV stabilised earlier and at lower overall levels, possibly reflecting greater surgeon familiarity with the dV interface and instrument articulation, as well as the cumulative transferable experience gained during prior CMR adoption. These findings highlight the complexity of comparing platforms without accounting for adoption sequence, surgeon exposure, and institutional learning [19]. 

Operative time variability appeared narrower with the dV system compared with both laparoscopy and CMR platforms, suggesting improved predictability that may enhance theatre scheduling and operational efficiency [20]. Although operative duration was longer with dV than with conventional laparoscopy in colonic resections, this may be partly attributable to the higher use of intracorporeal anastomosis in the robotic cohort, as randomised trials have demonstrated that this technique increases procedural duration compared with extracorporeal methods [20,21]. Nonetheless, intracorporeal anastomosis provides important clinical benefits, including faster return of bowel function, reduced postoperative pain, and fewer wound-related complications, without an associated increase in major morbidity [12, 20]. 

Oncological quality, measured by lymph node yield, showed a consistent trend favouring dV. This reached statistical significance in the full cohort and in rectal resections. Although differences of 3 to 4 nodes may not be clinically substantial at an individual level, higher yields are associated with improved staging accuracy and survival in population-level data. Previous comparative studies have also reported modest lymph node advantages with dV over laparoscopy [17] and small early series suggest potential improvements with emerging platforms [18]. Our real-world data reinforce that robotic platforms—particularly dV—perform at least equivalently to laparoscopy in oncological quality, with signals of improved yields in technically demanding pelvic cases [22]. 

Complication rates, including anastomotic leak, unplanned ICU admission, transfusion, and Clavien Dindo grade 2 or higher events, were broadly similar across approaches, with dV demonstrating numerically lower rates for most endpoints. Although differences did not consistently reach statistical significance after adjustment, the directionality of effect—particularly the lower leak and major-complication rates with dV—aligns with broader international experience [17]. Importantly, CMR showed higher complication rates in rectal cancer, with over half of patients experiencing Clavien Dindo grade 2 or higher complications in unadjusted analysis. This likely reflects early learning-curve effects rather than inherent platform limitations, as performance improved markedly over time. Nonetheless, these findings highlight the need for robust structured training, proctored early cases, and careful case selection during robotic rollout—core principles emphasised by the NICE EVA framework [16,19, 20,23].

Learning-curve analyses demonstrated clear operative learning with both platforms, with reductions in operating time and earlier plateauing for dV. CMR curves showed greater early variability and a more prolonged familiarisation phase, particularly for rectal resections, which is consistent with published early-adoption studies [18]. These data reinforce the value of cumulative robotic experience: surgeons with higher prior CMR case numbers appeared to transition more effectively to dV, supporting the concept that robotic skills retain cross-platform transferability. At centre level, staged adoption of multiple robotic systems may therefore accelerate platform-agnostic proficiency [7, 8]. 

Taken together, our findings contribute important NHS-specific comparative outcomes for two contemporary robotic systems relative to established laparoscopic practice. They directly align with NICE EVA priorities by providing real-world evidence on safety, perioperative value, workflow impact, and platform-specific learning behaviour. As multiple new robotic systems continue to enter global markets [18], comparative data such as these are essential to support procurement decisions, prevent fragmented implementation, and ensure equitable access to advanced minimally invasive surgery across the NHS [21].

This study has several important limitations. It reflects a real-world, non-randomised design, with the three cohorts corresponding to distinct chronological periods rather than contemporaneous comparison groups. Consequently, observed differences—particularly in length of stay—may in part reflect temporal changes in perioperative care, including maturation of ERAS pathways, evolving discharge and virtual ward practices, refinements in anaesthetic and postoperative management, and increasing institutional and multidisciplinary team experience over time. Although adjustment was performed for key measured confounders, residual confounding related to calendar time and unmeasured factors (such as case complexity, pelvic anatomy, and perioperative decision-making) cannot be excluded. The CMR cohort represents an early adoption phase, whereas the dV cohort benefitted from accumulated institutional, team, and surgeon experience, which may have influenced outcomes such as the observed learning-curve profiles. As such, causal attribution of outcome differences to surgical platform alone should be interpreted with caution. Sample sizes were also modest for subgroup analyses, particularly rectal resections, limiting statistical power. Nonetheless, the consistency of observed trends across multiple endpoints supports the validity of the findings while highlighting the need for contemporaneous comparative or time-adjusted analyses in future studies.

## Conclusion

In this real-world evaluation of sequential robotic adoption within an NHS colorectal service, both CMR and dV delivered safe oncological and perioperative outcomes comparable with conventional laparoscopy. dV was associated with shorter length of stay, higher lymph node yield, and lower conversion and complication rates, and demonstrated more favourable learning-curve behaviour, with earlier stabilisation of operative times. CMR performed safely but was associated with longer operating times and higher early complication rates in rectal cancer, largely reflecting early learning-curve effects.

These findings underscore the importance of structured training, staged implementation, and continuous performance monitoring during robotic rollout [19, 24]. They also provide contemporary evidence aligned with the NICE EVA remit, supporting informed procurement, service planning, and multi-platform robotic adoption strategies across the NHS 16. Further prospective evaluation, including formal cost-effectiveness analysis, is needed to determine the long-term system-level value of each platform within modern colorectal cancer pathways [11, 25].

## Data Availability

The data used in this study were derived from routine clinical care within an NHS Trust. Anonymised data may be requested from the corresponding author, subject to institutional approval.
